# Support for immunization registries among parents of vaccinated and unvaccinated school-aged children: a case control study

**DOI:** 10.1186/1471-2458-6-236

**Published:** 2006-09-22

**Authors:** Robert W Linkins, Daniel A Salmon, Saad B Omer, William KY Pan, Shannon Stokley, Neal A Halsey

**Affiliations:** 1 Thailand Ministry of Public Health - US CDC Collaboration, National Center For HIV, STD, and TB Prevention (previously at National Immunization Program), Centers for Disease Control and Prevention, Atlanta Georgia 30333, USA; 2 Department of Epidemiology and Health Policy Research, College of Medicine, University of Florida, 1329 SW 16th Street, Room 5239, Gainesville, Florida 32608, USA; 3 Department of International Health, Johns Hopkins Bloomberg School of Public Health, 615 N. Wolfe Street, Room W5041, Baltimore, Maryland 21205, USA; 4 National Immunization Program, Centers for Disease Control and Prevention, Mailstop E-05, 1600 Clifton Rd., NE, Atlanta, Georgia 30333, USA

## Abstract

**Background:**

Immunizations have reduced childhood vaccine preventable disease incidence by 98–100%. Continued vaccine preventable disease control depends on high immunization coverage. Immunization registries help ensure high coverage by recording childhood immunizations administered, generating reminders when immunizations are due, calculating immunization coverage and identifying pockets needing immunization services, and improving vaccine safety by reducing over-immunization and providing data for post-licensure vaccine safety studies. Despite substantial resources directed towards registry development in the U.S., only 48% of children were enrolled in a registry in 2004. Parental attitudes likely impact child participation. Consequently, the purpose of this study was to assess the attitudes of parents of vaccinated and unvaccinated school-aged children regarding: support for immunization registries; laws authorizing registries and mandating provider reporting; opt-in versus opt-out registry participation; and financial worth and responsibility of registry development and implementation.

**Methods:**

A case control study of parents of 815 children exempt from school vaccination requirements and 1630 fully vaccinated children was conducted. Children were recruited from 112 elementary schools in Colorado, Massachusetts, Missouri, and Washington. Surveys administered to the parents, asked about views on registries and perceived utility and safety of vaccines. Parental views were summarized and logistic regression models compared differences between parents of exempt and vaccinated children.

**Results:**

Surveys were completed by 56.1% of respondents. Fewer than 10% of parents were aware of immunization registries in their communities. Among parents aware of registries, exempt children were more likely to be enrolled (65.0%) than vaccinated children (26.5%) (p value = 0.01). A substantial proportion of parents of exempt children support immunization registries, particularly if registries offer choice for participation. Few parents of vaccinated (6.8%) and exempt children (6.7%) were aware of laws authorizing immunization registries. Support for laws authorizing registries and requiring health care providers to report to registries was more common among parents of vaccinated than exempt children. Most parents believed that the government, vaccine companies or insurance companies should pay for registries.

**Conclusion:**

Parental support for registries was relatively high. Parental support for immunization registries may increase with greater parental awareness of the risks of vaccine preventable diseases and utility of vaccination.

## Background

Immunizations have been tremendously successful in the United States, reducing childhood vaccine preventable diseases (VPDs) by 98–100% [1,2]. However, this impressive accomplishment does not guarantee future success: immunization coverage must be maintained to avoid resurgence of disease [3]. The immunization schedule has become increasingly complex as new vaccines have been introduced, making it difficult for parents and health care providers to remember the immunization schedule and comply with vaccine recommendations.

Immunization registries can help ensure high immunization coverage. As defined by the National Vaccine Advisory Committee (NVAC), immunization registries are "confidential, computerized information systems that contain information about immunizations and children" [4]. Immunization registries can be used for measuring vaccine coverage, generating reminders when immunizations are due and recalls when immunizations are overdue, identifying pockets of need for targeted interventions, improving vaccine safety by reducing over-immunization and calculating accurate denominators important for post-licensure vaccine safety studies, as well as many other important public health functions [4]. The federal government, states and non-profit organizations such as the Robert Wood Johnson Foundation's All Kids Count Program have invested substantial resources into the development of immunization registries, yet only 48% of U.S. children less than 6 years old are enrolled in a registry [5]. Only 76% of public and 39% of private vaccination provider sites reported administered immunizations to a registry in the last six months of 2004 [5]. Immunization registries must be fully operational and contain complete immunization records in order for their potential to be wholly realized.

Parental support for immunization registries has not been well characterized. Focus group research indicates that, although most parents are very positive about registries, they tend to follow their doctors' advice regarding participation [4]. The NVAC [4] and the Centers for Disease Control and Prevention [6] recommend that parents be given a choice about participating in immunization registries. The decision to participate can be "opt-out" (the registry is automatically populated using birth registry or other means and parents can choose to have all or part of their child's information removed) or "opt-in" (requires parental permission before any information about the child is entered into the registry). Opt-out registries are generally more efficient as it is easier to enter information at birth and then give parents the choice to remove the data rather than depending on an active response by parents.

Many states have laws to assist in the establishment and implementation of registries. As of October 2000, 24 of 51 jurisdictions (50 states and District of Columbia) had legislation authorizing the establishment of a registry, 12 mandated provider reporting to the registry, and 14 required explicit consent (opt-in) to be in a registry [8]. Parental support for these types of state registry laws has not been explored. Understanding parental support for immunization registries is important to registry development, as registries require substantial public funding and extremely high parental participation for their potential to be realized.

Much debate has surrounded who should pay for immunization registries [4]. Registry development, to date, has been financially supported by federal, state and local funding, private foundations, and managed care organizations [7]. During NVAC discussions, it was suggested that those who benefit from immunization registries should contribute to the cost of registry development and maintenance, including health care providers, vaccine companies and parents/patients [4].

The objectives of this study were: (1) to characterize support for immunization registries and laws authorizing registries and requiring providers to report to registries, and (2) to characterize parental preference for registry participation (opt-in and opt-out) and determine who they believe should pay for registries among parents of vaccinated and unvaccinated school-aged children.

## Methods

We conducted a case-control study of the parents of 815 children (cases) who were exempt (for any reason, including medical) for one or more vaccine antigens required by law for school entry and the parents of 1630 fully vaccinated children (controls). Two vaccinated control children in the same grade and school were randomly selected per case child. Children were recruited from 112 private and public elementary schools (grades kindergarten through 5) in Colorado (n = 25), Massachusetts (n = 23), Missouri (n = 34), and Washington (n = 30). Twenty exempt children were identified as siblings with different last names when the school addressed the envelopes and older siblings were removed from the study to avoid sending a duplicate survey to the same household. Study selection and recruitment methods have been described in detail elsewhere [9]. The states were selected based on the proportion of exemptions (low, medium and high compared with other states) and geographical distribution of states. Immunization registry attributes were not a consideration for state selection as the primary study purpose was to explore immunization exemption issues. This study was approved by the Committee on Human Research, Johns Hopkins Bloomberg School of Public Health.

### Survey content

Parents of exempt children were asked to verify that their child had not received one or more vaccines required for school entry and if the child received the complete or less-than-complete number of doses for each vaccine series. Parents who indicated that they did not have their child vaccinated for medical reasons were asked to indicate the medical condition that contraindicated vaccination. Respondents were provided with a brief description of immunization registries and asked to indicate if they support or oppose registries on a five point Likert scale ranging from "strongly oppose" to "strongly support"; if they were aware of a registry in their area and, if so, if their child was enrolled in the registry; and if they were aware of and if they would support laws authorizing immunization registries and requirements for health care providers to report to registries. Respondents were given a brief description of "opt-in" and "opt-out" and asked to indicate their preference to these options and whether having a choice regarding participation affected their support for registries. Respondents were informed that it costs about $4 per child per year to have an immunization registry [7,10], and were asked if they thought this was a good use of money and, if so, who should pay for immunization registries (parents, doctors, insurance companies, government, other, or not sure). Respondents were also asked to use a five point Likert scale to estimate the probability that an unimmunized child would contract a disease for which vaccines are recommended for elementary school children (polio, measles, mumps, rubella, diphtheria, pertussis, tetanus, *haemophilus influenzae *type b, hepatitis B, and varicella) during a ten year period ("impossible" to "very likely"); how serious it would be for an 8-year-old to develop one of these diseases ("not at all serious" to "very serious"); how effective the vaccines are in preventing children from getting these childhood diseases ("not at all protective" to "very protective"); and how safe the vaccine is ("dangerous" to "very safe"). Respondents were asked to self identify demographic characteristics, including age (9 categories, starting with 18–20 years and continuing by 5-year intervals with ≥ 61 as the highest), education (6 categories of grade completed: grade 4, grade 8, grade 12 or GED test, some college, college graduate, or postgraduate, and race or ethnicity (White, Hispanic, Black non-Hispanic, Native American, and other). Surveys took approximately 30 minutes to complete; a sample is available on-line [11].

### Data analysis

Parents were excluded from the primary data analysis if their child had been listed by the school as exempt but the parent indicated that their child was fully vaccinated, or if the parent provided a medical contraindication compatible with ACIP/AAP guidelines (based on review by the senior author).

Support for registries, laws authorizing registries, and laws requiring providers to report to registries were dichotomized into "strongly support" or "support" versus all other responses. Frequency of survey responses were calculated by state and were tested for differences in proportion answering each question affirmatively using the Fishers Exact Test. Frequency of survey responses were calculated for parents of vaccinated children and parents of exempt children. Odds ratios were used to compare differences in responses between parents of exempt (any antigen) and vaccinated children. In order to assess if survey responses (outcome variables) varied among parents of exempt children by the number of antigens a child was exempt for, exempt children were further categorized into 4 groups: exempt for 1 antigen, exempt for 2–5 antigens, exempt for 6–9 antigens and exempt for 10 antigens. The Cochran-Armitage test for trend was conducted across vaccinated children and children exempt for different numbers of antigens (vaccinated children and 4 groups described above) [12,13].

General constructs for respondents' assessments of disease susceptibility and severity, and vaccine efficacy and safety were created using the respondent's mean scores for all 10 antigens/diseases. These construct scores were dichotomized by the lower quartile among all respondents, indicating if a parent had "low" perceived disease susceptibility and severity and vaccine efficacy and safety. A logistic regression model was used to assess support for immunization registries (outcome variable) associated with low disease susceptibility and severity and low vaccine safety and efficacy (independent variables), adjusting for the vaccination status of the child (exempt versus vaccinated). Relationships between independent and dependent variables were generally consistent across states (data not presented) and consequently state data were combined.

## Results

Surveys were returned by 391(48.6%) of the 805 parents of exempt children and 976 (59.9%) of the 1630 parents of fully vaccinated children, for an overall response rate of 56.1%. Information on non-responders was not collected and consequently it is not possible to compare responders to non-responders.

Of the 391 parents of exempt children identified by the school, 86 reported that their children were fully vaccinated and an additional 28 provided valid medical contraindications for vaccination. These 114 children were dropped from the analysis. The remaining 277 parents of children with non-medical exemptions were included in the analyses; 33 (11.9%) children were exempt for 1 antigen, 73 (26.4%) children were exempt for 2–5 antigens, 57 (20.6%) children were exempt for 6–9 antigens, and 114 (41.1%) children were exempt for all 10 antigens.

The median age of respondents was 36–40 years and parents of exempt children tended to be a bit older than parents of vaccinated children. The median level of education for the study sample was some college, and parents of exempt children tended to be a bit more educated. The majority of vaccinated respondents (91.7%) and exempt respondents (94.5%) were white. The majority of surveys (88.5%) were completed by mothers.

Registry characteristics of the study states are summarized in Table 1. Fewer than 10% of parents were aware of immunization registries in their communities (Table 2), with a state range of 4.0% (Massachusetts – the only opt-in state) to 11.7% (Missouri). Respondents in Missouri and Washington were more likely to report being aware of a law authorizing an immunization registry compared with Colorado and Massachusetts (Table 2). Among parents aware of registries, exempt children were more likely to be enrolled in an immunization registry (65.0%) than vaccinated (26.5%) children (p value = 0.01). Few parents of vaccinated or exempt children were aware of laws authorizing immunization registries (6.8% and 6.7%, respectively). Support for laws authorizing registries and requiring health care providers to report to registries was more common among parents of vaccinated than exempt children, although parents with children exempt for only one antigen expressed similar support as parents of vaccinated children (Table 3). Support for registries, support for law authorizing registries, and support for law requiring providers to report to registries (Table 3) decreased with increasing numbers of exemptions, a statistically significant trend.

Parents of vaccinated children were more likely to support opt-out compared with opt-in registries and parents of exempt children were more likely to support opt-in compared with opt-out registries (Table 4). A statistically significant trend was identified among parents of exempt children for preference toward opt-in, preference toward opt-out, support for registries regardless of choice, and no support for registries despite choice. Providing a choice for participation in registries increased parental support among parents of exempt children, from 32.6% (Table 3) to 45.4% (Table 4), yet the availability of choice had little impact on support for registries among parents of vaccinated children. The majority of parents of vaccinated children (70.7%) and a substantial proportion of exempt parents (45.4%) supported immunization registries either because of or regardless of choice regarding registry participation (Table 4). Only 7.5% of parents of vaccinated children did not support registries despite choice, while more than 20% of parents of vaccinated children were unsure of their support for registries ("don't know"). Parents of vaccinated children were more likely than parents of exempt children to report that registries are worth $4 per year per child (Table 5). Most parents believed that the government, vaccine companies or insurance companies should pay for registries as opposed to parents (Table 5). Support for immunization registries was lower among parents with low perceived disease susceptibility (Odds Ratio (OR): 0.56; 95% Confidence Interval (CI) 0.39–0.81), disease severity (OR: 0.49; 95% CI 0.35–0.68), vaccine safety (OR: 0.51; 95% CI 0.36–0.74), and vaccine efficacy (OR: 0.63; 95% CI 0.44 – 0.91), adjusting for the child's vaccination status.

## Discussion

Most parents were not aware of immunization registries, including parents in Missouri and Washington, states that report greater than 75% of children are enrolled in registries. Parental support for registries was relatively high, particularly among parents of vaccinated children and parents of children with an exemption to only one antigen. A substantial proportion of parents of exempt children supported immunization registries, particularly if registries offered choice for participation, notable given that these parents refused one or more vaccines recommended for children. We were surprised to find that parents aware of registries were more likely to have their child enrolled in a registry if the child was exempt rather than vaccinated. Nearly a third of parents of vaccinated and exempt children did not indicate a preference for opt-in versus opt-out, suggesting that this difference may not be important to many parents.

The increased support for registries among parents of exempt children when participation is optional suggests that this may be an important registry attribute in gaining support for vaccine registries among parents who do not fully vaccinate their children. Yet, parental support for immunization registries may not be a strong predictor in registry participation given our study found that few parents were aware of registries even in states with high registry participation and most parents (even parents with antigen-specific exemptions) support registries. Greater efforts may be needed to make parents aware of registries to ensure that parents are properly informed. Anecdotal information suggests that only a small proportion of parents choose to opt-out or refuse to opt-in to immunization registries [7]. Our study findings suggest that opt-out may be a preferred strategy to opt-in given overall parental support for registries and the general efficiency of opt-in versus opt-out.

This study has the potential for non-response bias. If bias exists, the likely result is an overestimate of support for registries as parents who do not recognize the value of vaccines and immunization registries may have been less likely to complete vaccine related surveys. It is also possible that parents opposed to immunization registries were more motivated to respond. We were unable to compare child or parental characteristics between participants who completed surveys and those who refused, since no information was collected on refusals and the investigators were blinded to the names of respondents. Participation in immunization registries, registry types and registry laws vary by state and such differences may account for some of the differences in study finding by state. While the maturity of state registries and registry characteristics were not included in state selection criteria, the four states included in this study represent a rather broad range of registries (Table 1). Few parents were aware of registries which limits the generalizability of our findings. Parental reporting of enrollment may suffer from information bias as parents may have had their children enrolled in registries but were not aware of this. Schools were not selected based upon registry catchment area and consequently it was not possible to link parental responses with access to immunization registries. Care should be taken in generalizing from this study to the entire nation; parents were selected from only four states.

Associations found between support for immunization registries and perception of susceptibility to and severity of VPDs, and safety and efficacy of vaccines, suggest that parental support for immunization registries may increase with greater parental awareness of the risks of VPDs and utility of vaccination. For diseases that have been effectively controlled, many parents are not aware of the risks of disease [14].

Health care providers play an important role in parents' decision making about vaccine issues[15]; provider attitudes toward immunization registries likely affect parental attitudes toward immunization registries. Support for immunization registries by health care providers is critical for their success and many parents may rely on health care providers for determining participation in registries. In a recent study, the need to consolidate scattered vaccine records, closely followed by state mandated participation, was identified as the most influential reasons for provider registry participation [16]. Reasons for not participating in registries including too much resources, duplicate systems, lack of awareness of registry, incompatibility with existing data systems, confidentiality concerns, few pediatric patients, and insufficient technical support [16]. Provider participation in registries has been shown to vary by type of provider (pediatrician, family physician, nurse), type of provider practice (private practice, HMO, public or community clinic), size of practice, and community type (urban/rural) [17,18].

## Conclusion

Parents of vaccinated and unvaccinated school-aged children in the four states surveyed were largely unaware of the immunization registries in their region or state, even with reported child participation rates ranging from 7% to 81%. Despite this lack of awareness, the majority of parents interviewed were supportive of immunization registries, legislation authorizing immunization registries, and laws mandating provider reporting to registries. However, parents of exempt children were less like to be supportive of registries and registry legislation, and this lack of support increased as the number of vaccines their children were exempted for increased. Nonetheless, a substantial proportion of parents of exempt children supported immunization registries, particularly if registries offer choice for participation.

The usefulness of immunization registries correlates with the proportion of the childhood population enrolled as well as the quality of immunization data contained in the registry. Without high participation rates, targeted immunization interventions to areas of low immunization coverage will be of little value. Without complete and accurate immunization records, providers will understandably have little faith in registry information and ignore registry attributes of providing immunization decision support and generating messages to alert parents that their children are due or delinquent for a vaccine. With an increasingly complex immunization schedule, these attributes are becoming essential strategic elements to maintaining high immunization coverage levels.

## Competing interests

The author(s) declare that they have no competing interests.

## Authors' contributions

RL contributed to data analysis and interpretation, and manuscript development. DS contributed to study design, survey instrument development, data analysis and interpretation, and manuscript development. SO contributed to study design, data collection and analysis, and manuscript development. WP contributed to data analysis and interpretation, and manuscript development. SS contributed to study design and data interpretation. NH contributed to study design, survey instrument development, data analysis and interpretation, and manuscript development. All authors read and approved the final manuscript.

## Pre-publication history

The pre-publication history for this paper can be accessed here:



## Figures and Tables

**Table 1:** Immunization Registry characteristics of Colorado, Massachusetts, Missouri and Washington

**Registry Characteristic [19]**	**Colorado**	**Massachusetts**	**Missouri**	**Washington**
Catchment area	Regional	Regional	Statewide	Statewide
Provisions for participation	Opt-out	Opt-in	Opt-out	Opt-out
Populated by birth data?	Yes	No	Yes	Yes
Child participation level ^a^	43%	7%	81%	76%
% of public providers enrolled in registry	89%	84%	100%	86%
% of enrolled public providers submitting data within past 6 months	62%	67%	78%	99%
% of private providers enrolled in registry	30%	3%	22%	44%
% of enrolled private providers submitting data within past 6 months	57%	100%	38%	98%
				
**Registry Legislation [20]**				
State law authorizing immunization registry	Yes	No	No	No
State law mandate reporting?	No	N/A	N/A	N/A
Type of consent needed to enter the registry	Implied consent	Written consent	Implied consent	Implied consent
If implied consent, are there provisions to opt out or limit access to the registry?	No	N/A	No	Yes

^a ^Child participation level is defined as the proportion of children less than 6 years of age in the catchment area who are enrolled in the registry with 2 or more immunizations recorded.

**Table 2:** Frequency (Percent) of Parents who are Aware of and Support Immunization Registries (IRs) by State

	Colorado	Massachusetts	Missouri	Washington	Total	Fishers Exact Test^a ^P Value
Aware of IR	19 (5.6)	14 (4.0)	28 (11.7)	34 (11.5)	95 (7.7)	<0.01
If Aware of IR, Child Enrolled in IR	6 (31.3)	7 (50.0)	9 (32.1)	15 (45.0)	37 (38.2)	0.70
Support IR	232 (67.0)	212 (60.6)	151 (63.3)	189 (63.7)	784 (63.7)	0.42
Aware of Law Authorizing IR	17 (4.9)	12 (3.4)	24 (10.2)	30 (10.0)	83 (6.5)	<0.01
Support Law Authorizing IR	244 (70.2)	241 (68.7)	174 (73.0)	230 (77.7)	889 (72.2)	0.21
Support Law Requiring Providers to Report to IR	241 (69.5)	237 (67.7)	165 (69.2)	218 (73.5)	861 (70.0)	0.64

a = Fishers Exact Test is a chi square test that computes exact p values with no approximation.

**Table 3:** Frequency (Percent) of Parents Aware and Supportive of Immunization Registries (IRs) by Child's Vaccination Status

	Vaccinated	Exempt for Any Antigens	Unadjusted Odds Ratio and 95% CI^a,b^	Number of Antigens Exempt For			
				
				1	2–5	6–9	10
Aware of IR	66 (7.8)	18 (7.6)	0.96 (0.56–1.66)	1 (3.8)	7 (10.4)	4 (8.5)	6 (6.1)
If Aware of IR, Child Enrolled in IR	9 (26.5)	8 (65.0)	**5.14 **(1.68–15.71)	0 (0.0)	3 (42.9)	2 (66.7)	4 (75.0)
Support IR	652 (71.7)	80 (32.6)	**0.19 **(0.14–0.26)	21 (75.0)	26 (40.6)	11 (22.0)	20 (20.6)*
Aware of Law Authorizing IR	66 (6.8)	18 (6.7)	0.98 (0.57–1.67)	1 (3.2)	7 (9.6)	4 (7.3)	6 (5.4)
Support Law Authorizing IR	511 (81.6)	49 (32.0)	**0.11 **(0.07–0.16)	11 (84.6)	20 (46.5)	6 (18.2)	12 (18.8)*
Support Law Requiring Providers to Report to IR	494 (78.3)	55 (35.3)	**0.15 **(0.10–0.22)	8 (72.7)	19 (44.2)	11 (28.9)	17 (26.6)*

a: Odds Ratio compares parents of children exempt to any antigens to parents of vaccinated children. Interpretation of odd ratios: Parents of exempt children who were aware of registries had about 5 times as high odds of having their child enrolled in a registry compared with parents of vaccinated children who were aware of registries.

b: Odds Ratio in **Bold **P-Value < 0.05

* Trend test is statistically significant at <0.05

**Table 4:** Frequency (Percent) of Parental Preference for Opt-In vs. Opt-Out and Immunization Registry (IRs) Support by Child's Vaccination Status

	Vaccinated	Exempt for Any Antigens	Unadjusted Odds Ratio and 95% CI^a,b^	Number of Antigens Exempt For			
				
				1	2–5	6–9	10
Prefer Opt-In^c^	(32.7)	133 (50.0)	**2.06 **(1.55–2.71)	11 (35.5)	32 (44.4)	35 (63.6)	55 (50.9)*
Prefer Opt-Out^d^	342 (36.0)	54 (20.3)	**0.45 **(0.33–0.63)	10 (32.2)	19 (26.4)	6 (10.9)	19 (17.6)*
Don't Know	297 (31.3)	79 (29.7)	0.93 (0.69–1.25)	10 (32.3)	21 (29.2)	14 (25.5)	34 (31.5)
Total	950 (100.0%)	266 (100.0%)		31 (100.0%)	72 (100.0%)	55 (100.0%)	108 (100.0%)
Support IR regardless of choice	334 (34.7)	25 (9.3)	**0.18 **(0.12–0.28)	9 (30.0)	8 (11.3)	3 (5.3)	5 (4.5)*
Support IR because of choice	347 (36.0)	97 (36.1)	1.00 (0.76–1.33)	13 (43.3)	33 (46.5)	22 (38.6)	29 (26.1)
Do not support IR	72 (7.5)	76 (28.3)	**4.87 **(3.41–6.97)	2 (6.7)	16 (22.5)	20 (35.1)	38 (34.2)*
Don't Know	210 (21.8)	71 (26.3)	1.29 (0.95–1.77)	6 (20.0)	14 (19.7)	12 (21.0)	39 (35.2)*
Total	963 (100.0%)	269 (100.0%)		30 (100.0%)	71 (100.0%)	57 (100.0%)	111 (100.0%)

a: Odds Ratio compares parents of children exempt for any antigens to parents of vaccinated children. Interpretation of odd ratio: Parents of exempt children had about 3 times as high odds of preferring opt-in compared with parents of vaccinated children.

b: Odds Ratio in **Bold **P-Value < 0.05

* Trend test is statistically significant at <0.05

c: "opt-in" requires parental permission before any information about the child is entered into the registry

d: "opt-out" registry is automatically populated using birth registry or other means and parents can then have all or part of their child's information removed

**Table 5:** Frequency (Percent) of Parental Beliefs regarding Financial Worth and Responsibility of Immunization Registries (IRs), by Child's Vaccination Status

**Is IR worth $4/Child/Year**	**Vaccinated**	**Exempt**	**P-Value**
Yes	450 (47.1)	41 (15.3)	<0.01
No, Opposed to IR	73 (7.6)	87 (32.5)	<0.01
No, Cost Too Much	108 (11.3)	33 (12.2)	0.65
Don't Know	325 (34.0)	107 (40.0)	0.07
Total	956 (100.0%)	268 (100.0%)	
**Who Should Pay for IR ^**a**^**			
Government	302 (30.9)	40 (14.4)	<0.01
Vaccine Companies	245 (25.1)	49 (17.7)	0.01
Insurance Companies	211 (21.6)	21 (7.6)	<0.01
Parents	153 (15.7)	15 (5.4)	<0.01
Don't Know	89 (9.1)	26 (9.4)	0.89
Doctors	68 (7.0)	6 (2.2)	0.01

a: Not Mutually Exclusive

## References

[B1] Centers for Disease Control and Prevention (1999). Impact of vaccines universally recommended for children – United States, 1900–1998. JAMA.

[B2] Centers for Disease Control and Prevention (1999). Ten great public health achievements, 1900–1999: Impact of vaccines universally recommended for children. MMWR.

[B3] Gangarosa EJ, Galazka AM, Wolfe CR, Phillips LM, Gangarosa RE, Miller E, Chen RT (1998). Impact of the anti-vaccine movement on pertussis control: the untold story. Lancet.

[B4] National Vaccine Advisory Committee Development of community- and state-based immunization registries. http://www.hhs.gov/nvpo/report071100.pdf.

[B5] Centers for Disease Control and Prevention (2005). Immunization Information System Progress – United States, 2004. MMWR.

[B6] Centers for Disease Control and Prevention (2000). Community Immunization Registries Manual, Chapter II: Confidentiality. http://www.cdc.gov/nip/registry/pubs/cir-manual/cirman2.pdf.

[B7] Horne P, Saarlas K, Hinman A (2000). Costs of immunization registries. Experience form the All Kids Count II projects. Am J Prev Med.

[B8] Horlick G, Beeler S, Linkins R (2001). A review of state legislation related to immunization registries. Am J Prev Med.

[B9] Salmon DA, Moulton LH, Omer SB, DeHart P, Stokley S, Halsey NA (2005). Parental Factors Associated with Refusal of Childhood Vaccines: A Case-Control Study. Arch Pediatr Adolesc Med.

[B10] Linkins R, O'Carroll P, Yasnoff W, Ward E, Ripp L, Martin E (2002). Immunization registries – Critical tools for sustaining success. Public Health Informatics and Information Systems.

[B11] School Survey. http://www.vaccinesafety.edu/school-survey.htm.

[B12] Agresti A (1990). Categorical Data Analysis.

[B13] Margolin BH, Kotz S, Johnson NL (1988). Test for Trend in Proportions. Encyclopedia of Statistical Sciences.

[B14] Chen RT, Hibbs B (1998). Vaccine safety: current and future challenges. Pediatric Annals.

[B15] Taylor JA, Darden PM, Brooks DA, Hendricks JW, Baker AE, Wasserman RC (2002). Practitioner policies and beliefs and practice immunization rates: a study from pediatric research in office settings and the National Medical Association. Pediatrics.

[B16] Clark SJ, Cowan AE, Bartlett DL (2006). Private provider participation in satewide immunization registries. BMC Public Health.

[B17] Christakis DA, Stewart L, Bibus D, Stout JW, Zerr DM, MacDonald JK, Gale JL (1999). Provider's perceptions of an immunization registry. Am J Prev Med.

[B18] Gaudino JA, deHart MP, Cheadle A, Martic DP, Moore DL, Schwarz SJ, Schulman B (2002). Childhood immunization registries: gaps between knowledge and action from family practice physicians and pediatricians in Washington State, 1998. Arch Pediatr Adolesc Med.

[B19] Centers for Disease Control and Prevention 2004 Annual Progress Report. http://www2a.cdc.gov/nip/irar/grantee/granteeinfo.asp#grptg.

[B20] Centers for Disease Control and Prevention Survey of state immunization registry legislation. http://www.cdc.gov/nip/registry/privacy/legsurv.htm.

